# Metabarcoding of marine nematodes – evaluation of similarity scores used in alignment-based taxonomy assignment approach

**DOI:** 10.3897/BDJ.4.e10647

**Published:** 2016-11-15

**Authors:** Oleksandr Holovachov

**Affiliations:** ‡ Swedish Museum of Natural History, Stockholm, Sweden

**Keywords:** Nematoda, metabarcoding, BLAST, taxonomy assignment, 18S rRNA, OTU, alignment-based approach.

## Abstract

**Background:**

The diversity of organisms is being commonly accessed using metabarcoding of environmental samples. Reliable identification of barcodes is one of the critical steps in the process and several taxonomy assignment methods were proposed to accomplish this task, including alignment-based approach that uses Basic Local Alignment Search Tool (BLAST) algorithm. This publication evaluates the variability of 5' end of 18S rRNA barcoding region as expressed by similarity scores (alignment score and identity score) produced by BLAST, and its impact on barcode identification to family-level taxonomic categories.

**New information:**

In alignment-based taxonomy assignment approach, reliable identification of anonymous OTUs to supraspecific taxa depends on the correct application of similarity thresholds. Since various taxa show different level of genetic variation, practical application of alignment-based approach requires the determination and use of taxon-specific similarity thresholds.

## Introduction

Identification of anonymous barcodes clustered in Operational Taxonomic Units (OTUs) is one of the critical steps in metabarcoding studies of living organisms. It can be accomplished via several taxonomy-assignment tools belonging to four different categories: alignment-based, probabilistic, tree-based and phylogeny-based (Holovachov et al., unpublished). Alignment-based approach uses Basic Local Alignment Search Tool (BLAST, Altschul et al. 1990) algorithm implemented via NCBI server or as part of standalone software packages such as QIIME (Caporaso et al. 2010), LCAClassifier (Lanzén et al. 2012) or Taxonerator (Jones et al. 2011). The taxonomic placement of OTUs is based on whether the identity score (Bik et al. 2011, Bik et al. 2012, Creer et al. 2010, Fonseca et al. 2010, Fonseca et al. 2014, Gibson et al. 2015) or e-value (Sinniger et al. 2016) is above or below the predetermined similarity threshold. Overlap range between barcode and reference sequences can also be considered (Gibson et al. 2015). 90% cutoff for the identity score is most commonly used to assign taxonomy to anonymous OTUs using alignment-based approach with BLAST (Bik et al. 2011, Bik et al. 2012, Cowart et al. 2015, Creer et al. 2010, Fonseca et al. 2010, Fonseca et al. 2014) – OTUs having lower identity score are treated as unidentified, OTUs having higher identity score are identified to respective phyla. It is, however, not always clearly specified as to why OTUs with identity score lower than 90% are considered unidentifiable, and why identified OTUs (those that receive >90% identity score with reference sequence) are assigned only to the level of the phyla.

Recent publication describing Classification Resources for Environmental Sequence Tags (CREST, Lanzén et al. 2012) uses following similarity cutoffs to identify anonymous OTUs with LCAClassifier implementation of Megablast: 97% identity for genera, 95% for families, 90% for orders, 85% for classes and 80% for phyla. CREST reference databases include both Pocaryotic and Eucaryotic sequences, but similarity thresholds are based on the procaryotic 16S rRNA analysis of Cole et al. (2010), which defines 99% identity equal to species, 96.5% – genera, 90% – families, and 84% as equivalent to orders (or 1%, 3.5%, 10% and 16% difference per position) based on single linkage clustering. Another publication (Giongo et al. 2010) defines 99% sequence identity as equivalent to species, 95% – genera, 90% – classes/orders/families, and 80% – phyla, based on publications by Hong et al. (2005), Schloss and Handelsman (2004).

Barcoding regions are comparatively short and intentionally defined to include hypervariable domains, while the above mentioned rRNA similarity measures are based on comparison of full length sequences that also include highly conserved regions. Thus, similarity measures based on complete genes may or may not reflect variability of the barcoding regions. Moreover, variability of rRNA can be very different in closely related taxa (see the comparison of the families Cephalobidae and Panagrolaimidae below). Published similarity measures (Cole et al. 2010, Hong et al. 2005, Schloss and Handelsman 2004) themselves are based on distance calculations, and not on BLAST-derived scores. Therefore, similarity thresholds used in identification of metabarcodes need to be reevaluated and, if necessary, adjusted, using actual BLAST-based comparison of identity scores of the barcoding region for individual taxa. There is another issue that, to my knowledge, has not been specifically considered. While identifying own metabarcoding dataset (Haenel et al., in press, Holovachov et al., in press) I found that large number of reference sequences do not have complete overlap with the barcoding region of the 18S rDNA gene commony used for nematodes and other meiofauna (Holovachov 2016), and it is not clear how much impact does it have on the efficiency of the identification. Thus, the goal of this paper is to evaluate identity scores between barcode-sized sequences and reference dataset (often without 100% overlap) produced by BLAST search algorithm, and describe variability of these scores for species grouped in family-level taxonomic categories, as justified elsewhere (Holovachov et al. in press).

## Materials and Methods

### 1. Sequence data

SILVA database (Quast et al. 2012) is regularly used in metabarcoding studies to create reference dataset (Cowart et al. 2015, Lanzén et al. 2012, Lindeque et al. 2013, Haenel et al. in press). The entire Nematoda alignment of it was downloaded on December of 2015. At the first step, all sequences were manually checked in order to remove animal parasitic and exclusively terrestrial nematode species, sequences already known to be incorrectly identified, unidentified sequences (environmental sequences), and non-nematode sequences placed within Nematoda (see Holovachov 2016). The alignment was trimmed to the size of the barcoding region (see section 2 of Materials and Methods), only sequences that had 100% coverage with the barcoding region were retained for the comparison. All sequences (identified to species or genus level) from the following five predominantly marine nematode families were used in this study: Desmodoridae (represented by 21 taxa), Chromadoridae (30 taxa), Comesomatidae (12 taxa), Monhysteridae (21 taxa), Xyalidae (14 taxa) (Suppl. material 1). Two terrestrial families, Cephalobidae (represented by 16 taxa) and Panagrolaimidae (18 taxa), were also included for comparison (Suppl. material 1).

### 2. Barcoding region

This publication evaluates the variability of the barcoding region of the 18S rRNA gene that includes V1 and V2 variable regions (Holovachov 2016) and is used in barcoding and metabarcoding studies of nematodes in particular (Floyd et al. 2002) and of marine meiofauna in general (Fonseca et al. 2014, Fonseca et al. 2010, Mohrbeck et al. 2015, Sinniger et al. 2016, Haenel et al. in press, Holovachov et al., in press).

### 3. Analysis

Every barcode-size sequence was manually compared with reference sequences available in the Nucleotide collection (excluding uncultured/environmental sample sequences) of the NCBI database using BLASTN 2.5.0 search algorithm (Madden 2002). Two separate comparisons were done: in the first case all results were sorted by maximum score; in the second case only the results that produced 100% query cover were considered. Following three records were noted:

1. *Identity score* received by the *nearest ingroup taxon* (sequence from the same family), i.e., the closest scoring match from the same family that is not the same sequence;
2. *Identity score* received by the *furthest ingroup taxon* before the first outgroup taxon (sequence from the different family), i.e., the furthest scoring match from the same family immediately preceding the closest scoring match that belongs to a different family;
3. *Identity score* received by the *nearest outgroup taxon* (sequence from the different family), i.e., the closest scoring match from a different family.

Standard statistical measures (minimum, maximum, averade and standard deviation) were calculated for alignment score, identity score and coverage when appropriate (Suppl. materials 2, 3) and used for comparison below. Certain sequences were ignored during sorting, these include unidentified sequences, environmental and uncultured sequences that were not automatically excluded during BLAST search, and two misidentified sequences (GQ503078 *Monhystera* sp. and KJ636248 *Mononchus aquaticus*).

## Results

### 1. Variable coverage

The results of BLAST searches are summarized in Suppl. material 2. The lowest identity scores for the nearest ingroup taxon varied considerably in marine families, from 98% in the family Comesomatidae to 91% in the family Monhysteridae, while average values were more consistent across families (97.8-99.3%). This alone shows that 95% similarity threshold used to define families in LCAClassifier of CREST (Lanzén et al. 2012) may in some cases be too strict and may exclude potentially identifiable sequences.

**Figure 1.**
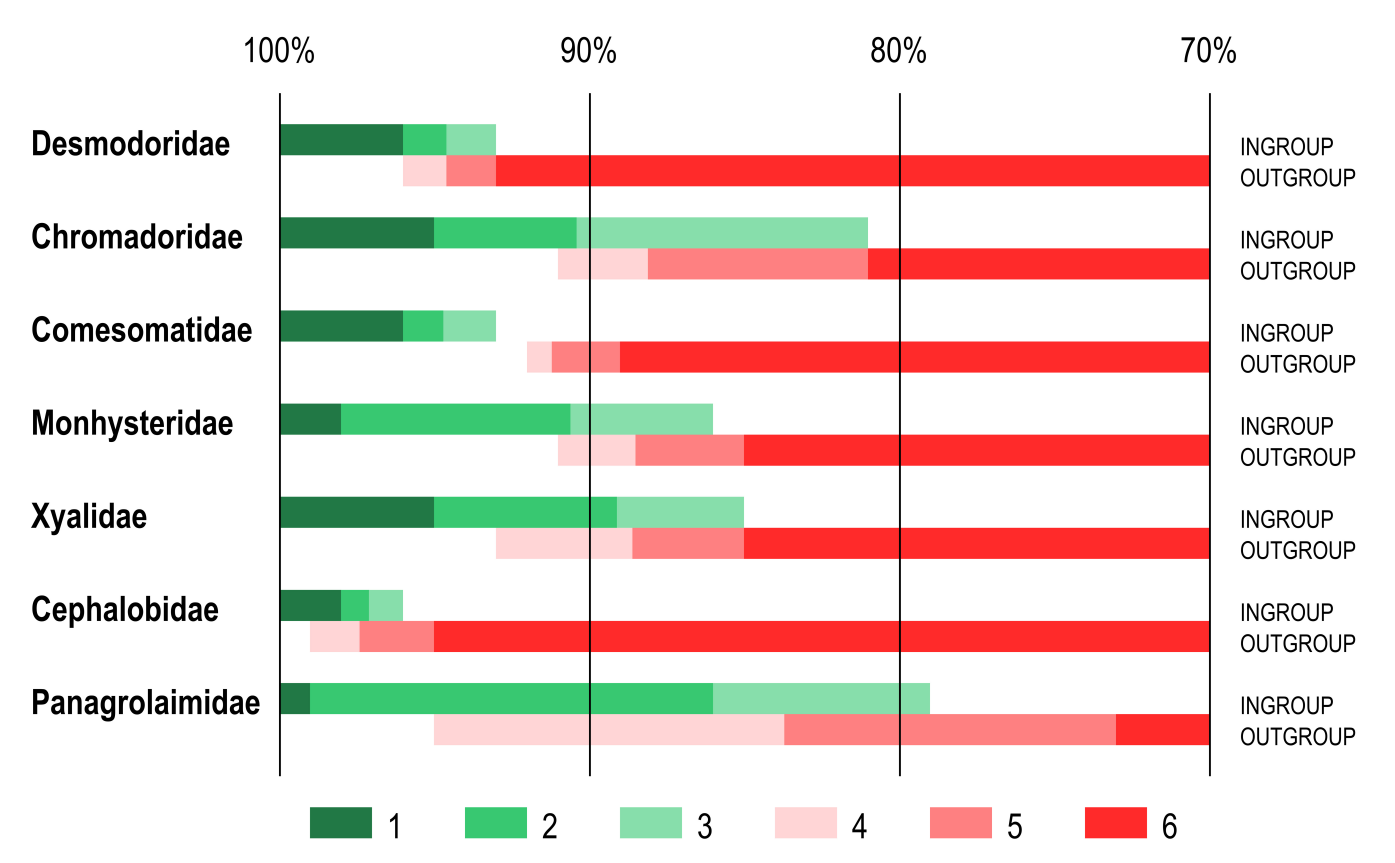
Ranges of identity scores of furthest ingroup taxon and nearest outgroup taxon as revealed by BLAST comparison of query sequences with reference dataset with variable coverage between sequences. Ranges of identity scores. 1 – 100% identity to maximum identity score of the furthest ingroup taxon; 2 – maximum to average identity scores of the furthest ingroup taxon; 3 – average to minimum identity scores of the furthest ingroup taxon; 4 – maximum to average identity scores of the nearest outgroup taxon; 5 – average to minimum identity scores of the nearest outgroup taxon; 6 – minimum identity score of the nearest ingroup taxon to 70% identity threshold.

Identity scores for the furthest ingroup taxon and nearest outgroup taxon also varied considerably between different families (Fig. 1, Suppl. material 2). Furthest ingroup taxon for the families Desmodoridae and Comesomatidae showed relatively narrow range, 93-96% identity score to query sequence, while same scores for the families Chromadoridae, Monhysteridae and Xyalidae varied between 81-86% (lowest) and 95-98% (highest). Identity scores for the nearest outgroup taxon were also variable, with the highest 93-96% in Desmodoridae and the lowest 81-91% in Chromadoridae. For comparison, in the family Cephalobidae identity scores for the furthest ingroup taxon range within 96-98% and for the nearest outgroup taxon – within 95-99%. Same values in the family Panagrolaimidae are 79-99% (furthest ingroup taxon) and 73-95% (nearest outgroup taxon). What is more important is that ranges of identity scores for furthest ingroup taxon and nearest outgroup taxon showed considerable overlap for all compared families (marine and terrestrial) except for the family Comesomatidae (Fig. 1).

These two terrestrial families purposely chosen for comparison also present two specific challenges that were not seen in marine families. For example, in the family Cephalobidae in many cases the nearest outgroup taxon with lesser coverage of 94% would receive higher identity score (98-99% identity) than the furthest ingroup taxon with 100% coverage (96-97% identity). The family Panagrolaimidae presented a different challenge – all compared barcodes of the genus *Halicephalobus* received very low sequence coverage with the furthest ingroup (32%) and nearest outgroup (17-27%) taxa, and found no outgroup sequences with 100% coverage, even though many sequences in the reference database have full overlap with them. This can indicate that BLAST algorithm has difficulties aligning highly modified sequences of *Halicephalobus*.

### 2. 100% Coverage

The results of BLAST searches are summarized in Suppl. material 3. The lowest identity scores for the nearest ingroup taxon varied considerably in marine families, from 98% in the family Comesomatidae to 86% in the family Chromadoridae, while average values were more consistent across families (97.0-99.2%) and very similar to the results described in the previous section (Results 1. Variable coverage).

**Figure 2.**
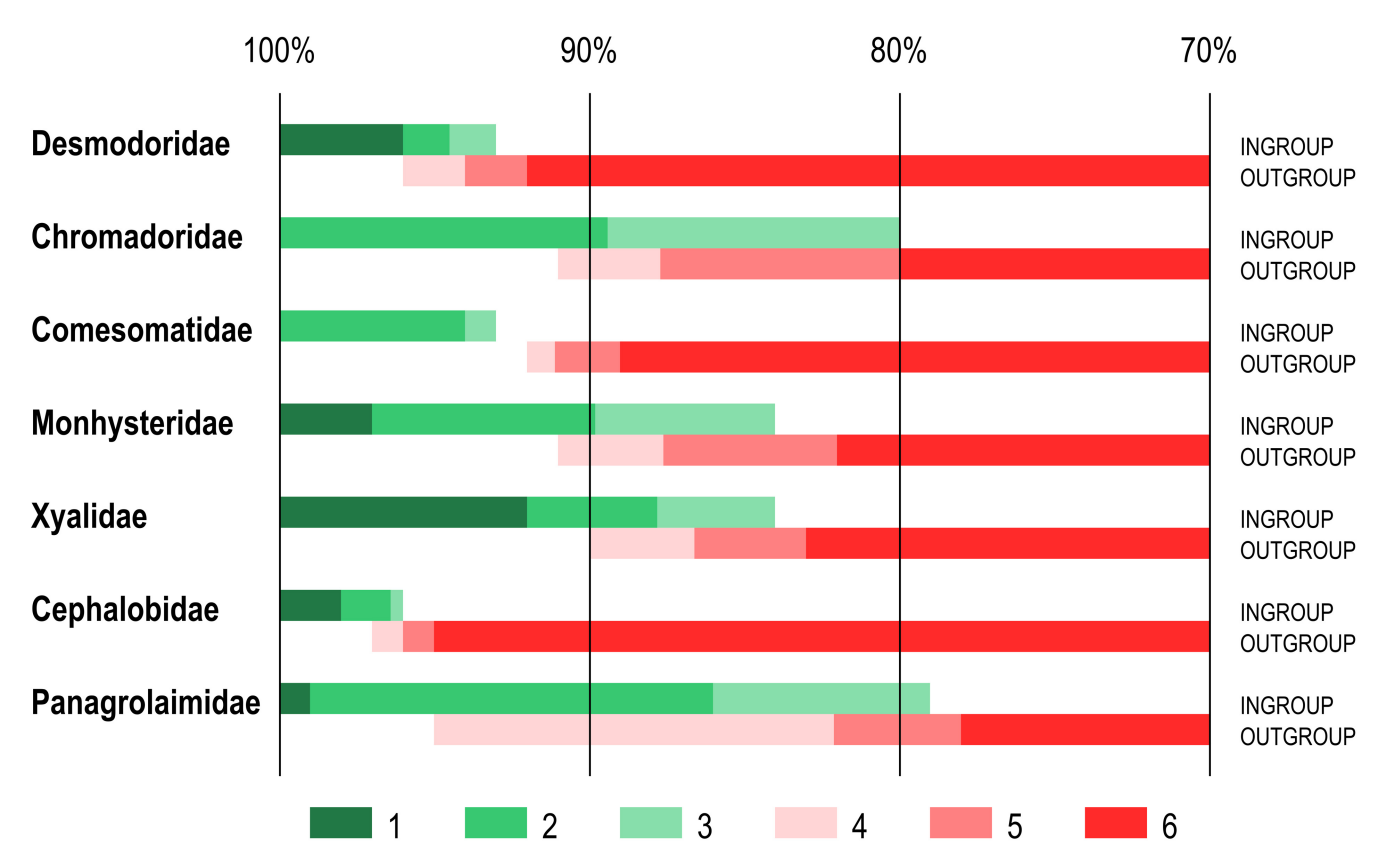
Ranges of identity scores of furthest ingroup taxon and nearest outgroup taxon as revealed by BLAST comparison of query sequences with reference dataset with 100% coverage between sequences. anges of identity scores. 1 – 100% identity to maximum identity score of the furthest ingroup taxon; 2 – maximum to average identity scores of the furthest ingroup taxon; 3 – average to minimum identity scores of the furthest ingroup taxon; 4 – maximum to average identity scores of the nearest outgroup taxon; 5 – average to minimum identity scores of the nearest outgroup taxon; 6 – minimum identity score of the nearest ingroup taxon to 70% identity threshold.

Identity scores for the furthest ingroup taxon and nearest outgroup taxon again showed considerable variation between different families (Fig. 2, Suppl. material 3). Furthest ingroup taxon for the family Desmodoridae showed relatively narrow range, 93-96% identity score to query sequence. For the remaining five families the same scores varied between 80-93% (lowest) and 92-100% (highest). 100% identity scores for the furthest ingroup taxon were noted in several cases and were caused by limited number of reference taxa that had 100% coverage with query sequence. Identity scores for the nearest outgroup taxon were also variable, with the highest 92-96% in Desmodoridae and the lowest 80-91% in Chromadoridae. For comparison, in the family Cephalobidae identity scores for the furthest ingroup taxon range within 96-98% and for the nearest outgroup taxon – within 95-97%. Same values in the family Panagrolaimidae are 79-99% (furthest ingroup taxon) and 78-95% (nearest outgroup taxon). Similarly to the preceding comparison (Results 1. Variable coverage) the ranges of identity scores for furthest ingroup taxon and nearest outgroup taxon showed considerable overlap for all families (marine and terrestrial) except for the family Comesomatidae (Fig. 2).

Limiting searches to sequences with 100% overlap affected two specific issues with the families Cephalobidae and Panagrolaimidae described above (Results 1. Variable coverage). In the family Cephalobidae the nearest outgroup taxon no longer have higher identity score than the furthest ingroup taxon (for same query sequence), making identifiication more reliable. In the case of the family Panagrolaimidae, limiting searches to sequences with 100% overlap produced no nearest outgroup hits for the genus *Halicephalobus*.

## Discussion

### Similarity thresholds

Only in one out of five analyzed families of marine nematodes, there was no overlap in ranges of identity scores between furthest ingroup taxon and nearest outgroup taxon. Remaining four marine and two terrestrial families showed considerable overlap between both values (identity score of the furthest ingroup taxon and identity score of the nearest outgroup taxon). Moreover, both values showed substantially different variability ranges and average values depending on the taxon in-question. It suggests that universal similarity thresholds applied to nematodes need to be used with great caution.

Even considering only highest scoring hits of the BLAST searches for alignment-based identification of OTUs should be done with great care. Due to scarcity of nematode reference dataset, many highest scoring hits have very low identity scores, especially in case when only 100% overlapping sequences are considered. In this analysis, nearest ingroup scores for some sequences from the family Chromadoridae were as low as 86%, thus using 95% or even 90% similarity threshold to assign anonymous OTUs to families will treat such cases unidentifiable. This problem can not be solved by broadening similarity cutoffs, as it will increase incorrect taxon assignment for all families, but only by filling in the gaps in the reference databases by specifically targeting those species and genera for which no sequence data is available.

### Impact of sequence coverage

Level of overlap between query and reference sequence has certain impact on identity scores in particular and on the identification process in general. While performing BLAST searches, I noticed numerous cases when outgorup taxa with lower coverage received higher identity scores than ingroup taxa with more complete coverage. On the other hand, limiting BLAST searches to sequences with only 100% coverage effectively limits the range of reference taxa to compare with – as already described in Holovachov (2016), large number of nematode sequences in GenBank are missing a substantial section from the 5' end of this particular barcoding region of rRNA gene.

### Problematic sequences

Presence of erroneous sequences in reference databases and its impact on identification of anonymous OTUs had been extensively discussed and illustrated (Blaxter et al. 2016, Schnell et al. 2015). In addition to several erroneous sequences discussed previously (Holovachov 2016), two more incorrectly identified sequences were found during blast searches: GQ503078 *Monhystera* sp. groups within the family Xyalidae instead of the family Monhysteridae, while KJ636248 *Mononchus aquaticus* groups within the family Monhysteridae instead of the family Mononchidae.

## Conclusions

The diversity of nematodes is seriously underrepresented in reference databases used for identification of anonymous barcodes (OTUs). When using alignment-based taxonomy assignment tools to identify nematode OTUs, it is important to know both (1) the lowest similarity thresholds that can be confidently applied to assign OTUs to supraspecific taxa, in order to maximize the efficiency of identification; and (2) the highest similarity thresholds that can ensure minimum number of mis-assigned OTUs.

Targeted sequencing of reference taxa from underrepresented nematode families is expected to improve the efficiency of alignment-based taxonomy assignment approach. Two groups of taxa should be specifically considered: (1) those species that are completely missing from the reference databases, and (2) those species, which sequences (already available in reference databases) do not have full coverage with the barcoding region used in metabarcoding studies.

It is also important to understand that universal similarity thresholds can only be applied with great caution, that taxon-specific similarity thresholds may be more effective to use, and that other taxonomy assignment methods may be more reliable for a particular dataset (Holovachov et al., in press).

## Supplementary Material

Supplementary material 1 Table S1. GenBank accession numbers and classification of sequences used in present analysis. Data type: list
File: oo_110493.pdf Holovachov

Supplementary material 2 Table S2. Variability of maximum alignment score and identity score for the nearest ingroup, furthest ingroup and nearest outgroup taxa in the BLASTN searches with variable coverage for each of five marine (Desmodoridae, Chromadoridae, Comesomatidae, Monhysteridae and Xyalidae) and two terrestrial (Cephalobidae and Panagrolaimidae) families of nematodes. Number of analyzed sequences for each family is given in parenthesis. Data type: list
File: oo_110494.pdf Holovachov

Supplementary material 3 Table S3. Variability of maximum alignment score and identity score for the nearest ingroup, furthest ingroup and nearest outgroup taxa in the BLASTN searches with 100% coverage for each of five marine (Desmodoridae, Chromadoridae, Comesomatidae, Monhysteridae and Xyalidae) and two terrestrial (Cephalobidae and Panagrolaimidae) families of nematodes. Number of analyzed sequences for each family is given in parenthesis. Data type: list
File: oo_110495.pdf Holovachov
