# Supplementary material for: Metabarcoding of marine nematodes – evaluation of similarity scores used in alignment-based taxonomy assignment approach
Source: Biodivers Data J. 2016 Nov 15;(4):e10647. doi: 10.3897/BDJ.4.e10647 (PMC5136674; doi:10.3897/BDJ.4.e10647)
Supplement: Supplementary material 1 — Table S1. GenBank accession numbers and classification of sequences used in present analysis. [file biodiversity_data_journal-4-e10647-s001.pdf]

File name: S1-TAXA.pdf

Table S1. GenBank accession numbers and classification of sequences used in present analysis.

Acc. number	Family	Genus	Species
Y16912	Desmodoridae	*Catanema*	sp.
AF047891	Desmodoridae	*Chromadoropsis*	*vivipara*
AY854215	Desmodoridae	*Desmodora*	*communis*
Y16913	Desmodoridae	*Desmodora*	*ovigera*
Y16915	Desmodoridae	*Eubostrichus*	*dianeae*
Y16916	Desmodoridae	*Eubostrichus*	*parasitiferus*
Y16917	Desmodoridae	*Eubostrichus*	*topiarius*
KF453617	Desmodoridae	*Eubostrichus*	sp.
KF453618	Desmodoridae	*Eubostrichus*	sp.
Y16918	Desmodoridae	*Laxus*	*cosmopolitus*
Y16919	Desmodoridae	*Laxus*	*oneistus*
KJ414468	Desmodoridae	*Leptonemella*	*vicina*
Y16920	Desmodoridae	*Leptonemella*	sp.
AY854216	Desmodoridae	*Metachromadora*	*remanei*
AF036595	Desmodoridae	*Metachromadora*	sp.
EF591339	Desmodoridae	*Metachromadora*	sp.
FJ040469	Desmodoridae	*Metachromadora*	sp.
Y16921	Desmodoridae	*Robbea*	*hypermnestra*
AY854217	Desmodoridae	*Spirinia*	*parasitifera*
Y16922	Desmodoridae	*Stilbonema*	*majum*
Y16923	Desmodoridae	*Xyzzors*	sp.
AY854204	Chromadoridae	*Atrochromadora*	*microlaima*
AY854205	Chromadoridae	*Chromadora*	*nudicapitata*
AY854206	Chromadoridae	*Chromadora*	sp.
KJ636220	Chromadoridae	*Chromadorina*	*bioculata*
KJ636221	Chromadoridae	*Chromadorina*	*bioculata*
AY854207	Chromadoridae	*Chromadorina*	*germanica*
KJ636256	Chromadoridae	*Chromadorina*	sp.
FJ040471	Chromadoridae	*Chromadorina*	sp.
FJ040470	Chromadoridae	*Chromadorina*	sp.
FJ969119	Chromadoridae	*Chromadorita*	*leuckarti*
KJ636254	Chromadoridae	*Chromadorita*	*leuckarti*

Acc. number	Family	Genus	Species
KJ636214	Chromadoridae	*Chromadorita*	*leuckarti*
FJ040473	Chromadoridae	*Chromadorita*	cf. *leuckarti*
AY854208	Chromadoridae	*Chromadorita*	*tentabundum*
JN968224	Chromadoridae	*Chromadorita*	*tentabundum*
AY854209	Chromadoridae	*Dichromadora*	sp.
FJ040506	Chromadoridae	*Dichromadora*	sp.
JN968222	Chromadoridae	*Dichromadora*	sp.
AY854210	Chromadoridae	*Neochromadora*	sp.
JN968215	Chromadoridae	*Neochromadora*	sp.
JN968255	Chromadoridae	*Neochromadora*	sp.
JN968230	Chromadoridae	*Neochromadora*	sp.
JN968246	Chromadoridae	*Neochromadora*	sp.
JN968267	Chromadoridae	*Neochromadora*	sp.
EF591341	Chromadoridae	*Prochromadora*	sp.
JN968223	Chromadoridae	*Punctodora*	*ratzeburgensis*
JN968227	Chromadoridae	*Punctodora*	*ratzeburgensis*
JN968283	Chromadoridae	*Punctodora*	*ratzeburgensis*
KJ636232	Chromadoridae	*Punctodora*	*ratzeburgensis*
AY854211	Chromadoridae	*Spilophorella*	*paradoxa*
AY854234	Comesomatidae	*Sabatieria*	*celtica*
EF591335	Comesomatidae	*Sabatieria*	*pulchra*
FJ040466	Comesomatidae	*Sabatieria*	*pulchra*
JN968250	Comesomatidae	*Sabatieria*	*pulchra*
JN968228	Comesomatidae	*Sabatieria*	*pulchra*
AY854236	Comesomatidae	*Sabatieria*	*punctata*
AY854237	Comesomatidae	*Sabatieria*	*punctata*
AY854235	Comesomatidae	*Sabatieria*	*punctata*
AY854239	Comesomatidae	*Sabatieria*	sp.
JN968221	Comesomatidae	*Sabatieria*	sp.
AY854240	Comesomatidae	*Setosabatieria*	*hilarula*
JN968273	Comesomatidae	*Setosabatieria*	*hilarula*
AJ966482	Monhysteridae	*Diplolaimella*	*dievengatensis*
AF036611	Monhysteridae	*Diplolaimelloides*	*meyli*
AF036644	Monhysteridae	*Diplolaimelloides*	*meyli*
AY593937	Monhysteridae	*Eumonhystera*	*filiformis*

Acc. number	Family	Genus	Species
KJ636238	Monhysteridae	*Eumonhystera*	*filiformis*
KJ636219	Monhysteridae	*Eumonhystera*	*filiformis*
KJ636239	Monhysteridae	*Eumonhystera*	*filiformis*
KJ636240	Monhysteridae	*Eumonhystera*	cf. *filiformis*
KJ636237	Monhysteridae	*Eumonhystera*	cf. *hungarica*
KJ636250	Monhysteridae	*Eumonhystera*	cf. *vulgaris*
EF591334	Monhysteridae	*Geomonhystera*	*villosa*
FJ040465	Monhysteridae	*Geomonhystera*	sp.
KJ636213	Monhysteridae	*Geomonhystera*	sp.
AJ966485	Monhysteridae	*Halomonhystera*	*disjuncta*
HF572952	Monhysteridae	*Halomonhystera*	sp.
FJ969130	Monhysteridae	*Monhystera*	*paludicola*
KJ636258	Monhysteridae	*Monhystera*	cf. *paludicola*
AY593938	Monhysteridae	*Monhystera*	*riemanni*
KJ636259	Monhysteridae	*Monhystera*	*stagnalis*
KJ636246	Monhysteridae	*Monhystera*	cf. *stagnalis*
AJ966507	Monhysteridae	*Tridentulus*	sp.
AY854223	Xyalidae	*Daptonema*	*hirsutum*
AY854224	Xyalidae	*Daptonema*	*normandicum*
AY854225	Xyalidae	*Daptonema*	*oxycerca*
AF047889	Xyalidae	*Daptonema*	*procerus*
AY854226	Xyalidae	*Daptonema*	*setosum*
JN968233	Xyalidae	*Daptonema*	*setosum*
FJ040463	Xyalidae	*Daptonema*	sp.
AJ966491	Xyalidae	*Metadesmolaimus*	sp.
AJ966505	Xyalidae	*Theristus*	*acer*
AY284693	Xyalidae	*Theristus*	*agilis*
AY284695	Xyalidae	*Theristus*	*agilis*
FJ040464	Xyalidae	*Theristus*	sp.
JN968231	Xyalidae	*Theristus*	sp.
KC920423	Xyalidae	*Zygonemella*	*striata*
AY284671	Cephalobidae	*Acrobeles*	*complexus*
AY284673	Cephalobidae	*Acrobeloides*	*apiculatus*
EU543174	Cephalobidae	*Acrobeloides*	*buetschlii*
EU196016	Cephalobidae	*Acrobeloides*	*maximus*

Acc. number	Family	Genus	Species
EU306344	Cephalobidae	*Acrobeloides*	*maximus*
AY284672	Cephalobidae	*Acrobeloides*	*nanus*
DQ102707	Cephalobidae	*Acrobeloides*	*nanus*
EU543175	Cephalobidae	*Acrobeloides*	*thornei*
AY284663	Cephalobidae	*Cephalobus*	*persegnis*
AY284662	Cephalobidae	*Cephalobus*	*persegnis*
AY284677	Cephalobidae	*Chiloplacus*	*propinquus*
AY284664	Cephalobidae	*Eucephalobus*	cf. *oxyuroides*
AY284666	Cephalobidae	*Eucephalobus*	*striatus*
AY284670	Cephalobidae	*Heterocephalobus*	*elongatus*
AY284668	Cephalobidae	*Heterocephalobus*	*elongatus*
AY284675	Cephalobidae	*Zeldia*	sp.
AF202156	Panagrolaimidae	*Halicephalobus*	*gingivalis*
JX674039	Panagrolaimidae	*Halicephalobus*	*gingivalis*
HQ697250	Panagrolaimidae	*Halicephalobus*	cf. *gingivalis*
JF706244	Panagrolaimidae	*Halicephalobus*	cf. *gingivalis*
GQ918144	Panagrolaimidae	*Halicephalobus*	sp.
AF083007	Panagrolaimidae	*Panagrellus*	*redivivus*
AF036599	Panagrolaimidae	*Panagrellus*	*redivivus*
AOMH01000003	Panagrolaimidae	*Panagrellus*	*redivivus*
AF202153	Panagrolaimidae	*Panagrobelus*	*stammeri*
FJ969134	Panagrolaimidae	*Panagrobelus*	*stammeri*
AJ567385	Panagrolaimidae	*Panagrolaimus*	*davidi*
AY284681	Panagrolaimidae	*Panagrolaimus*	*subelongatus*
EU543176	Panagrolaimidae	*Panagrolaimus*	*detritophagus*
KF011488	Panagrolaimidae	*Panagrolaimus*	*facetus*
KF011489	Panagrolaimidae	*Panagrolaimus*	cf. *papillosus*
DQ285636	Panagrolaimidae	*Panagrolaimus*	cf. *rigidus*
EU543179	Panagrolaimidae	*Procephalobus*	sp.
AF202165	Panagrolaimidae	*Turbatrix*	*aceti*
