# Supplementary material for: Metabarcoding of marine nematodes – evaluation of similarity scores used in alignment-based taxonomy assignment approach
Source: Biodivers Data J. 2016 Nov 15;(4):e10647. doi: 10.3897/BDJ.4.e10647 (PMC5136674; doi:10.3897/BDJ.4.e10647)
Supplement: Supplementary material 2 — Table S2. Variability of maximum alignment score and identity score for the nearest ingroup, furthest ingroup and nearest outgroup taxa in the BLASTN searches with variable coverage for each of five marine (Desmodoridae, Chromadoridae, Comesomatidae, Monhysteridae and Xyalidae) and two terrestrial (Cephalobidae and Panagrolaimidae) families of nematodes. Number of analyzed sequences for each family is given in parenthesis. [file biodiversity_data_journal-4-e10647-s002.pdf]

File name: S2-VAR.pdf

Table S2. Variability of maximum alignment score and identity score for the nearest ingroup, furthest ingroup and nearest outgroup taxa in the BLASTN searches with variable coverage for each of five marine (Desmodoridae, Chromadoridae, Comesomatidae, Monhysteridae and Xyalidae) and two terrestrial (Cephalobidae and Panagrolaimidae) families of nematodes. Number of analyzed sequences for each family is given in parenthesis.

Family	Alignment score	Identity score (%)	Coverage (%)
DESMODORIDAE (n=21)			
Nearest ingroup taxon	613.6±38.8 (562-673)	97.8±1.8 (95-100)	98.1±3.6 (90-100)
Furthest ingroup taxon	553.9±21.9 (521-590)	94.6±1.1 (93-96)	99.6±0.7 (98-100)
Nearest outgroup taxon	550.3±21.6 (520-582)	94.6±1.1 (93-96)	98.0±1.3 (97-100)
CHROMADORIDAE (n=30)			
Nearest ingroup taxon	635.9±34.4 (540-667)	98.8±1.6 (94-100)	98.9±3.1 (88-100)
Furthest ingroup taxon	424.3±72.3 (254-496)	90.4±3.6 (81-95)	90.0±7.7 (59-100)
Nearest outgroup taxon	416.4±69.3 (254-486)	87.9±3.2 (81-91)	99.3±2.0 (90-100)
COMESOMATIDAE (n=12)			
Nearest ingroup taxon	658.1±11.7 (632-671)	99.3±0.7 (98-100)	99.6±0.8 (98-100)
Furthest ingroup taxon	501.2±21.4 (470-538)	94.7±1.2 (93-96)	88.9±1.1 (87-90)
Nearest outgroup taxon	487.0±21.0 (455-514)	91.2±1.0 (89-92)	99.9±0.3 (99-100)
MONHYSTERIDAE (n=21)			
Nearest ingroup taxon	624.1±40.8 (479-654)	98.2±1.9 (91-100)	99.3±2.4 (89-100)
Furthest ingroup taxon	463.7±59.6 (370-593)	90.6±3.2 (86-98)	98.3±3.1 (89-100)
Nearest outgroup taxon	427.0±30.1 (361-486)	88.5±1.7 (85-91)	98.5±2.8 (93-100)
XYALIDAE (n=14)			
Nearest ingroup taxon	635.9±40.5 (534-669)	98.7±1.8 (93-100)	98.4±3.6 (90-100)
Furthest ingroup taxon	438.4±51.0 (370-516)	89.1±3.1 (85-95)	97.6± 5.0 (85-100)
Nearest outgroup taxon	429.1±47.7 (361-494)	88.6±2.5 (85-93)	97.6±2.9 (91-100)
CEPHALOBIDAE (n=16)			
Nearest ingroup taxon	660.8±15.4 (630-675)	99.2±0.8 (98-100)	99.9±0.3 (99-100)
Furthest ingroup taxon	603.0±17.0 (571-621)	97.1±0.6 (96-98)	97.6±3.0 (92-100)
Nearest outgroup taxon	600.3±17.4 (568-619)	97.4±1.6 (95-99)	96.3±3.0 (94-100)
PANAGROLAIMIDAE (n=18)			
Nearest ingroup taxon	609.7±84.0 (359-675)	97.0±3.8 (85-100)	98.7±2.3 (92-100)
Furthest ingroup taxon	290.5±185.8 (95-658)	86.0±6.5 (79-99)	71.2±30.1 (30-100)
Nearest outgroup taxon	248.8±158.2 (68-564)	83.7±5.7 (73-95)	74.1±33.4 (17-100)
