# Supplementary material for: Metabarcoding of marine nematodes – evaluation of similarity scores used in alignment-based taxonomy assignment approach
Source: Biodivers Data J. 2016 Nov 15;(4):e10647. doi: 10.3897/BDJ.4.e10647 (PMC5136674; doi:10.3897/BDJ.4.e10647)
Supplement: Supplementary material 3 — Table S3. Variability of maximum alignment score and identity score for the nearest ingroup, furthest ingroup and nearest outgroup taxa in the BLASTN searches with 100% coverage for each of five marine (Desmodoridae, Chromadoridae, Comesomatidae, Monhysteridae and Xyalidae) and two terrestrial (Cephalobidae and Panagrolaimidae) families of nematodes. Number of analyzed sequences for each family is given in parenthesis. [file biodiversity_data_journal-4-e10647-s003.pdf]

File name: S3-100.pdf

Table S3. Variability of maximum alignment score and identity score for the nearest ingroup, furthest ingroup and nearest outgroup taxa in the BLASTN searches with 100% coverage for each of five marine (Desmodoridae, Chromadoridae, Comesomatidae, Monhysteridae and Xyalidae) and two terrestrial (Cephalobidae and Panagrolaimidae) families of nematodes. Number of analyzed sequences for each family is given in parenthesis.

Family	Alignment score	Identity score (%)
DESMODORIDAE (n=21)		
Nearest ingroup taxon	608.6±43.9 (527-673)	97.0±2.1 (93-100)
Furthest ingroup taxon	552.6±20.7 (516-582)	94.5±1.1 (93-96)
Nearest outgroup taxon	547.8±22.1 (510-582)	94.0±1.2 (92-96)
CHROMADORIDAE (n=30)		
Nearest ingroup taxon	620.9±62.8 (370-667)	97.8±3.0 (86-100)
Furthest ingroup taxon	449.4±93.5 (261-667)	89.4±4.5 (80-100)
Nearest outgroup taxon	414.0±70.4 (243-486)	87.7±3.4 (80-91)
COMESOMATIDAE (n=12)		
Nearest ingroup taxon	657.0±13.9 (632-671)	99.2±0.8 (98-100)
Furthest ingroup taxon	541.5±42.4 (510-669)	94.0±2.0 (93-100)
Nearest outgroup taxon	485.0±21.9 (455-514)	91.1±1.1 (89-92)
MONHYSTERIDAE (n=21)		
Nearest ingroup taxon	617.8±53.6 (475-654)	97.8±2.6 (91-100)
Furthest ingroup taxon	455.4±76.9 (346-612)	89.8±3.8 (84-97)
Nearest outgroup taxon	416.9±39.7 (307-486)	87.6±2.1 (82-91)
XYALIDAE (n=14)		
Nearest ingroup taxon	627.4±54.9 (496-669)	97.7±2.8 (91-100)
Furthest ingroup taxon	419.6±50.9 (337-497)	87.8±2.4 (84-92)
Nearest outgroup taxon	398.4±47.5 (329-466)	86.6±2.4 (83-90)
CEPHALOBIDAE (n=16)		
Nearest ingroup taxon	659.9±15.5 (630-675)	99.1±0.7 (98-100)
Furthest ingroup taxon	601.9±10.1(586-625)	96.4±0.6 (96-98)
Nearest outgroup taxon	593.9±14.6 (568-619)	96.0±0.8 (95-97)
PANAGROLAIMIDAE (n=18)		
Nearest ingroup taxon	632.5±50.2 (503-673)	97.7±2.3 (92-100)
Furthest ingroup taxon	438.9±171.8 (233-658)	88.5±8.2 (79-99)
Nearest outgroup taxon	306.1±143.4 (209-564)	82.1±7.1 (78-95)
